# Non-contact measurement of glucose in urine by smartphone-based laser refractometry for diabetes monitoring

**DOI:** 10.1038/s41598-025-13946-9

**Published:** 2025-08-19

**Authors:** Amirul Badiuzzaman Sinin, Khairul Fikri Tamrin, Muhammad Hamdi Mahmood

**Affiliations:** 1 Faculty of Engineering, Universiti Malaysia Sarawak (UNIMAS), Kota Samarahan, Sarawak, 94300 Malaysia; 2 Faculty of Medicine and Health Sciences, Universiti Malaysia Sarawak (UNIMAS), Kota Samarahan, Sarawak, 94300 Malaysia

**Keywords:** Laser optical measurement, Refractometer, Non-invasive, Glucose monitoring, Smartphone, Medical research, Engineering, Optics and photonics

## Abstract

Diabetes mellitus is a global health issue affecting millions of people and requires regular glucose level monitoring. Current non-invasive methods, such as urinalysis (including colorimetry and biosensors), are primarily laboratory-based and lack user-friendliness, limiting their practicality for continuous glucose monitoring. Although promising, research on smartphone-integrated laser refractometry for glucose detection remains limited. To address this gap, a non-contact, smartphone-based laser refractometer for glucose monitoring was developed. This prototype measures the refractive index of urine by analyzing the refracted length of a laser line, which correlates with fasting blood glucose concentrations. The system uses a smartphone to capture high-resolution images of the laser line generated by total internal reflection through a rod and refraction through the urine sample. Assessments were conducted using controlled glucose concentrations, varying turbidity levels, different samples volume, and shelf-life conditions. Volumetric and shelf-life variations showed no significant impact on results, whereas turbidity assessments revealed a limitation of up to 57 nephelometric turbidity units (NTU). Fasting glucose levels measured from using the developed system were compared with laboratory fasting blood glucose results, yielding a correlation coefficients of 0.89 and a sensitivity of 4.8 mg/dL. The system is low-cost, making it accessible and suitable for telemedicine applications, offering remote glucose monitoring for patients. This approach paves the way for clinically relevant glucose detection in diabetic patients without the need for invasive finger-prick blood sampling.

**Supplementary Information:**

The online version contains supplementary material available at 10.1038/s41598-025-13946-9.

## Introduction

Diabetes mellitus is a well-known chronic metabolic disorder that can be categorized into three types: Type-1, Type-2 and gestational^1^. It is estimated that 90% of diabetic patients have Type 2 diabetes^2^. Type 1 is mainly caused by the destruction of pancreatic β-cells by the autoimmune system, and it can also occur at a young age due to genetics factors^1,3^. Of many, Type-2 is the most common, caused by defective insulin secretion and the body’s unresponsiveness to insulin, often due to an unhealthy lifestyle and poor diet^4^. Gestational diabetes, on the other hand, occurs during pregnancy due to carbohydrate intolerance^5^.

When a person is diagnosed with diabetes, it is crucial for them to monitor their glucose levels to maintain a healthy range and avoid complications such as kidney damage, stroke, and heart disease^6^. The problem with diabetes is that approximately 50% of patients remain undiagnosed^6^. This becomes extremely life-threatening, as these individuals are unaware of their condition and may continue an unhealthy lifestyle. This issue is especially prevalent in rural areas, where access to proper healthcare is limited.

Diabetes monitoring can be done frequently through semi-invasive or non-invasive methods, which include microwave sensing, spectroscopy, electrochemical analysis, fluorescence, and urinalysis. Microwave-based studies^7–11^ measure the permittivity of different body parts using sensor patches that require regular replacement. Spectroscopy methods include Raman, capacitance, and infrared techniques. Raman spectroscopy^12–15^ uses a Raman spectrometer to analyze spectra obtained from various body parts, where specific peaks are correlated with glucose levels. Capacitance spectroscopy^16–18^ measures the capacitance of body parts or samples by placing them between two sensors connected to a multimeter. Infrared spectroscopy involves passing infrared light through a sample and measuring the absorbance. Although these methods often result in high correlation coefficients with glucose levels, the devices involved used are typically complex and expensive. Electrochemical methods^19–23^ use glucose oxidase to catalyze the oxidation of glucose, producing hydrogen peroxide, which is then electrochemically detected to generate a signal correlated with glucose levels in interstitial fluid. Dehydrogenase enzymes, such as glucose dehydrogenase (GDH), can also be used in place of glucose oxidase to catalyze glucose oxidation. These enzymes use coenzymes such as flavin adenine dinucleotide (FAD)^24^ and nicotinamide adenine dinucleotide (NAD⁺)^25^ as electron acceptors. The reduced forms of these coenzymes (e.g., NADH) can be detected electrochemically, generating signals that correlate with glucose concentrations in interstitial fluid.

In many fluorescence-based glucose sensors, glucose oxidase is first used to catalyze the oxidation of glucose, producing hydrogen peroxide. The generated hydrogen peroxide then interacts with nanomaterials, such as silver nanocubes^26^, or carbon nanoparticles^27^, modulating their fluorescence properties, which are detected using a spectrofluorometer.

Urinalysis includes colorimetry and biosensors for measuring glucose, protein, creatinine, albumin, and more. In colorimetric urinalysis, test strip color changes are analyzed^28,29^, while other studies have focused on the colorimetry of gold nanoparticles^30,31^. Tohl et al.^30^ measured creatine and albumin via test strip color changes, achieving a detection limit of 1 mg/dL and a correlation coefficient of 0.73. However, stability was an issue, as urine color variation affected the accuracy of the results. Similarly, Pohanka and Zakova^31^ proposed a simpler setup to measure test strip colorimetry for glucose detection, with a detection limit of 1.98 mg/dL, although a 60-second incubation was necessary for optimal results. Feng et al.^28^ investigated the colorimetry of gold nanoparticles (AuNPs), achieving highly sensitive glucose detection (0.0002–0.0018 mg/dL) but at the cost expense of a slow detection time (190 s) and expensive biosensor fabrication. Firdaus et al.^29^ also studied AuNP-based colorimetry for glucose detection, achieving high sensitivity (0.00008 mg/dL), though it required optimal sample conditions (pH 7, temperature 30 °C, and 30-minute incubation) and involved complex and costly biosensors fabrication. Although colorimetry is inherently non-invasive, the results are qualitative in nature.

Detection of glucose using novel biosensors has also been proposed. For instance, Hajimiri et al.^32^ measured the response of glucose oxidase in a novel solution under optimal conditions (pH 7.4, temperature 60 °C and mixed for 5 min in a vortex mixer), achieving a detection limit of 0.103 mg/dL. Nguyen et al.^33^ used glucose-spiked solutions instead of real urine, utilizing zinc sulfide as a biosensor, and achieved a detection limit of 2.3 mg/dL. Fenoy et al.^34^, on the other hand, used graphene field-effect transistors with glucose oxidase, obtaining a detection limit of 0.0007 mg/dL, but at the expense of a longer assay time (190 s) and a complex fabrication process.

In this study, we have developed a smartphone-based laser refractometer that uses only urine samples and a smartphone to measure the refractive index of urine. This eliminates the need for disposable materials and complex instruments, potentially enabling telemedicine applications in rural settings. Table 1 compares the performance of the smartphone-based laser refractometer with previous colorimetric research. For instance, test strip-based colorimetry in^31^ achieved an R^2^ of 0.998 and a sensitivity of 1.98 mg/dL. Meanwhile, colorimetry using AuNP, as reported in^28^ and^29^, yielded R^2^ values of 0.876 and 0.998 and sensitivities of 0.0002 and 0.00008 mg/dL, respectively. These results show that AuNP-based colorimetry offers superior detection limits compared to test strip colorimetry. However, the correlation coefficient obtained in this study is relatively high and compares favourably with other urinalysis methods, many of which have yet to be validated in practical applications. As turbidity is the primary limitation in the current study, image processing techniques such as Wiener deconvolution will be explored to correct turbidity-induced image degradation. This is expected to improve the accuracy of image analysis, which will be addressed in our forthcoming manuscript.

**Table 1:** Comparison of previous Urinalysis methods for glucose sensing.

Method of urinalysis	Limit of detection (mg/dL)	Incubation time	Correlation Coefficient, *R*^2^	Ref.
Colorimetry of test strips	1.98	60 s	0.998	^31^
Colorimetry of AuNPs	0.0002	190 s	NA	^28^
Colorimetry of AuNPs	0.00008	30 min	0.99	^29^
Biosensors (GOx)	0.103	5 min	0.99	^32^
Biosensors (ZnS)	2.3	-	NA	^33^
Biosensors (GOx)	0.0007	190 s	0.95	^34^
Smartphone-based laser refractometer	4.8	-	0.89	Present work

## Results and discussion

### Preliminary experiment

**Fig. 1:**
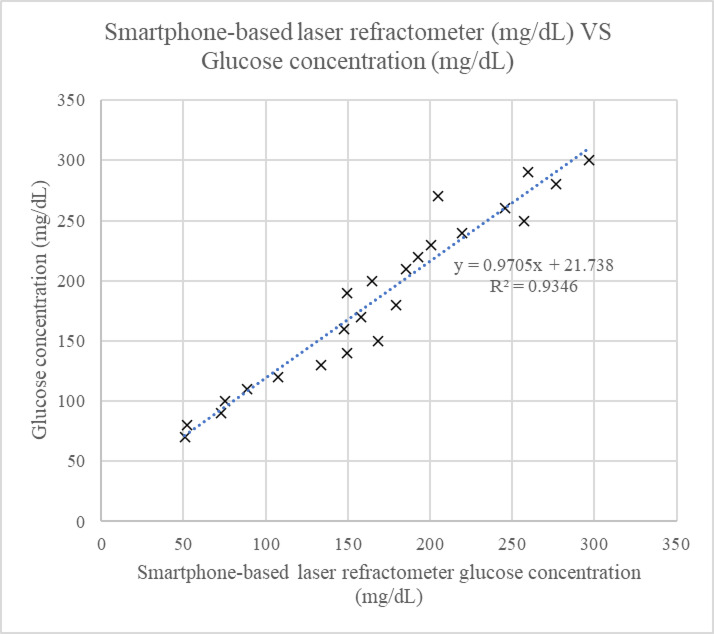
Results of smartphone-based laser refractometer glucose concentration (mg/dL) VS with glucose concentration (mg/dL) of prepared samples.

**Fig. 2:**
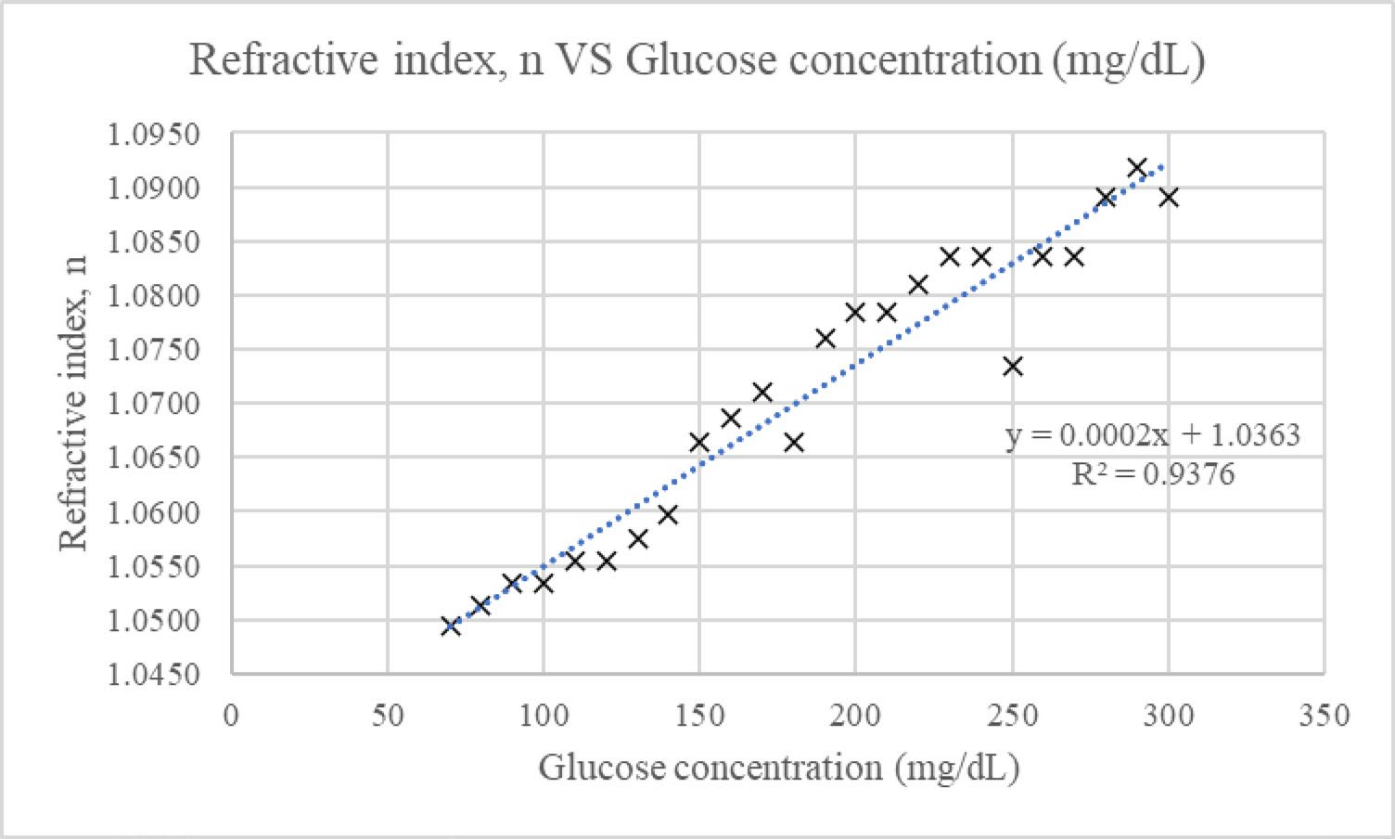
Results of refractive index, n VS glucose concentration (mg/dL).

The first phase of experimentation was done using glucose samples (70–300 mg/dL) prepared by mixing food grade glucose powder with water. The refractive index of the glucose samples was measured by observing the refracted laser line in the prepared solutions. Results from this phase demonstrated an inverse relationship between glucose concentration and the length of the laser line. Other studies have also reported a similar trend, where glucose levels exhibit an inverse relationship with the measured parameters, such as permitivity in microwave methods^7–11^ and voltage in capacitance methods^16–18^. As shown in Fig. 1, the correlation coefficient between the proposed method and the glucose samples was R^2^ = 0.93, with a sensitivity of 0.971 mg/dL. For each glucose concentration, three images were acquired, and the average angle was calculated to measure the respective refractive index^35^. The results in Fig. 2 confirmed that a higher glucose concentration corresponds to a higher refractive index with a standard deviation ranging from 0 to 2.88 (refer to **Appendix A** for detailed mean and standard deviation for each measurement).

### Assessing the effects of different urine volumes

**Fig. 3:**
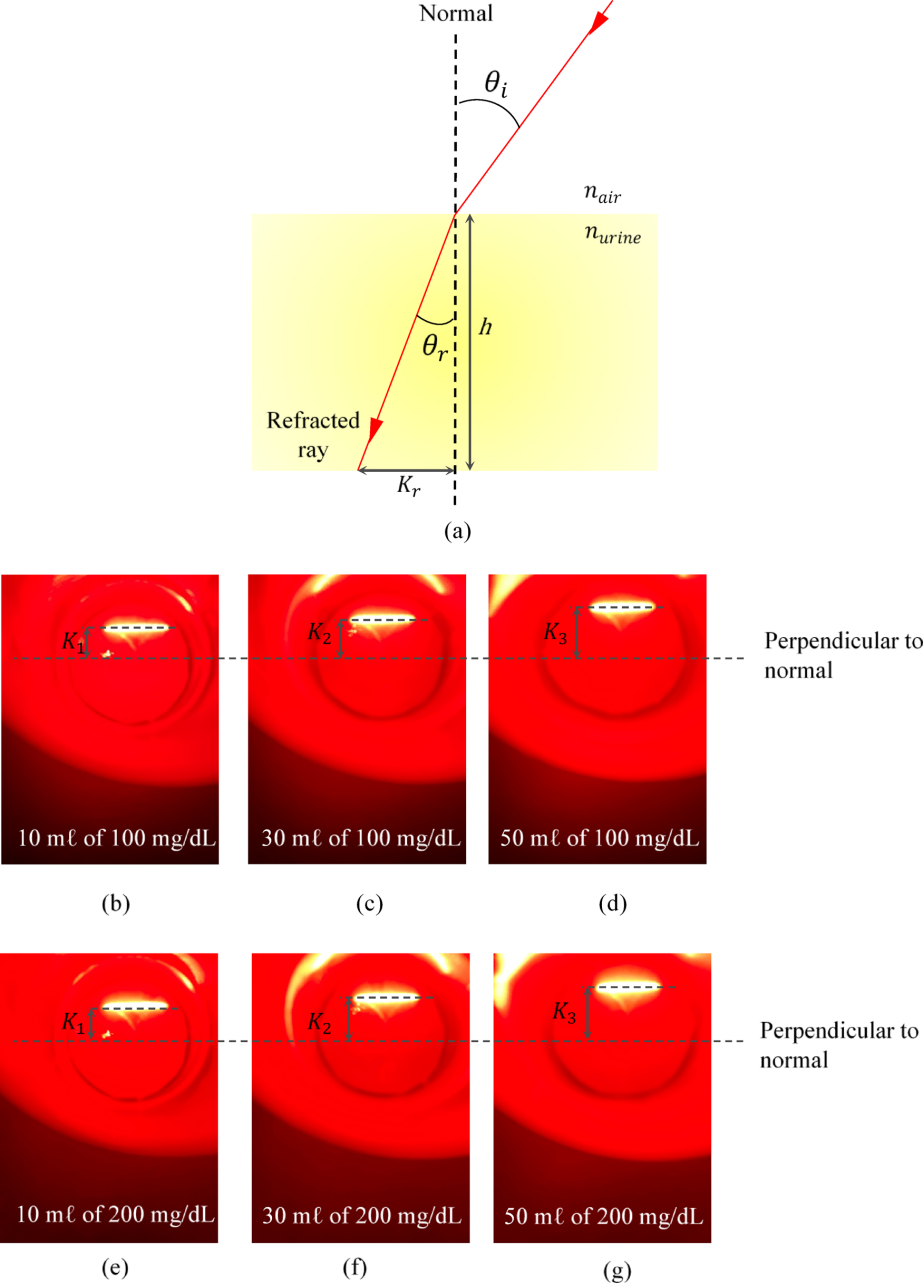
(**a**) Detailed side view of sample with higher glucose concentration. Results of (**b**) 10 mℓ of 100 mg/dL, (**c**) 30 mℓ of 100 mg/dL, (**d**) 50 mℓ of 100 mg/dL, (**e**) 10 mℓ of 200 mg/dL, (**f**) 30 mℓ of 200 mg/dL, and (**g**) 50 mℓ of 200 mg/dL glucose solutions.

To account for variations in human urine volume, a volumetric effect study was also performed. Because maintaining consistent urine volumes is laborious and time-consuming, this calibration used three different concentrations and three different volumes to assess any effects. It is noteworthy that an average void of human urine is 224 mℓ^36^, which is more than sufficient for the purpose of this device. This assessment was conducted by varying the sample volumes while maintaining the same glucose concentration, in order to understand the effect of sample depths on the measurement results. Based on Table 2, varying the sample volumes does not affect the results. This can be explained using Fig. 3.

**Table 2:** Results of varying volume and varying glucose levels on measured refracted laser line.

Volume (mℓ)	Concentration of prepared glucose solution		
	100 (mg/dℓ)	200 (mg/dℓ)	300 (mg/dℓ)
10	804 pixels	740 pixels	678 pixels
20	806 pixels	743 pixels	680 pixels
30	805 pixels	739 pixels	680 pixels
40	803 pixels	738 pixels	677 pixels
50	806 pixels	737 pixels	678 pixels
Mean	804.8 pixels	739.4 pixels	678.6 pixels
Std dev	1.30	2.30	1.34

Using trigonometry, the relationship can be expressed as:

1 $$\:{\theta\:}_{r}={{tan}}^{-1}\left(\frac{{K}_{r}}{h}\right)$$

where $\:{K}_{r}$ is the position of the refracted laser line on the base of the container, and $\:h$ is the liquid depth of the sample from the base of the container.

By rearranging Eqs. (1) and (2), it is shown that any variation in liquid depth affects only the planar position of the refracted laser on the container base, in proportion to the liquid depth, provided that the same container is used, as described in Eq. (3). In other words, $\:{K}_{r}$ is directly proportional to $\:h$, since $\:{\theta\:}_{r}$ remains the same for different volumes of the same liquid with the same refractive index.

2 $$\:\frac{{n}_{air}}{{n}_{urine}}=\frac{\text{sin}{\theta\:}_{r}}{\text{sin}{\theta\:}_{i}}$$

3 $$\:{n}_{urine}=\frac{{n}_{air}\text{sin}{\theta\:}_{i}}{\text{sin}\left[{\text{tan}}^{-1}\left(\frac{{K}_{r}}{h}\right)\right]}$$

### Assessing the effects of different urine turbidity

**Fig. 4:**
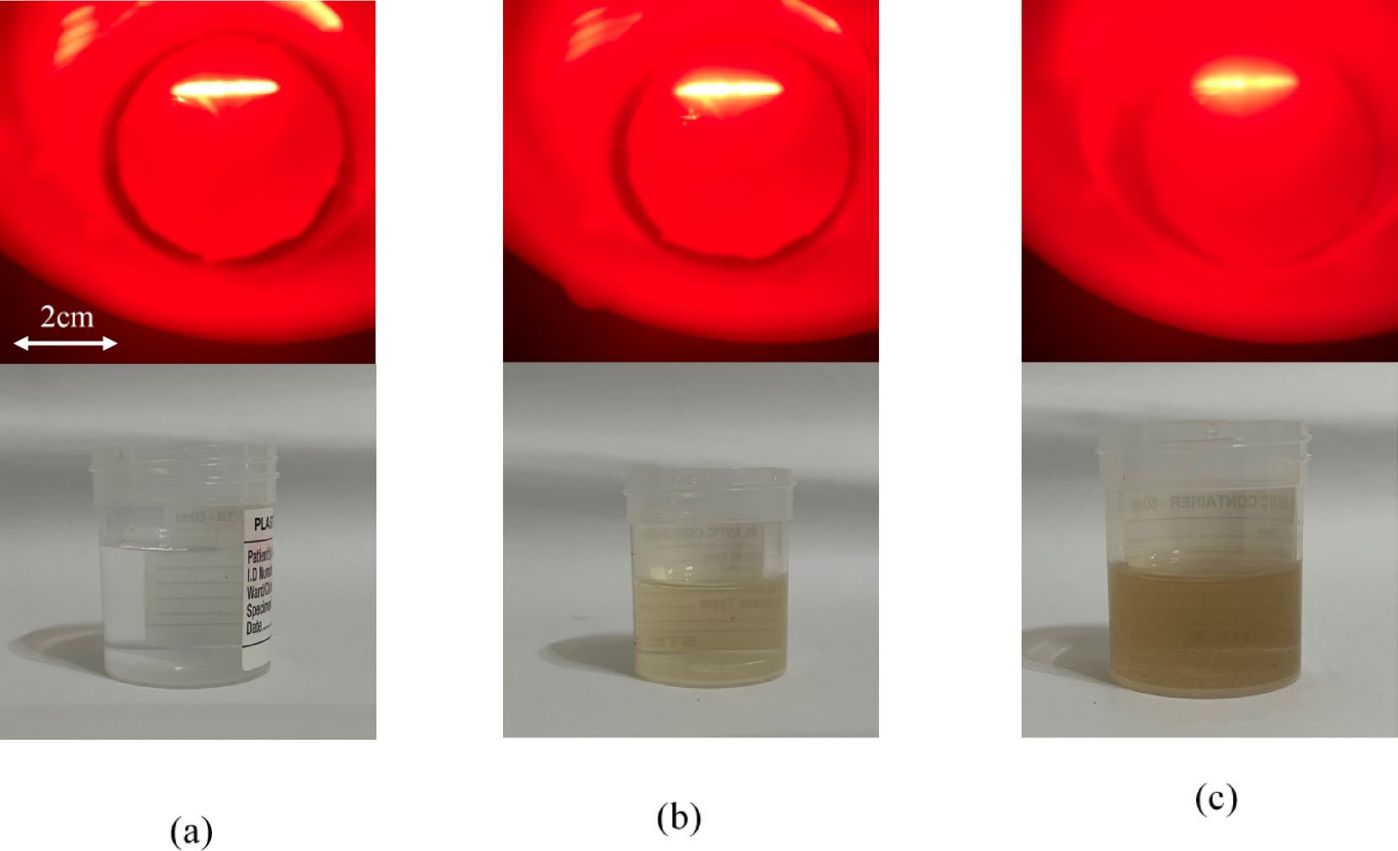
Image of sample with turbidity of (**a**) 0 NTU, (**b**) 57 NTU, and (**c**) 106 NTU.

**Fig. 5:**
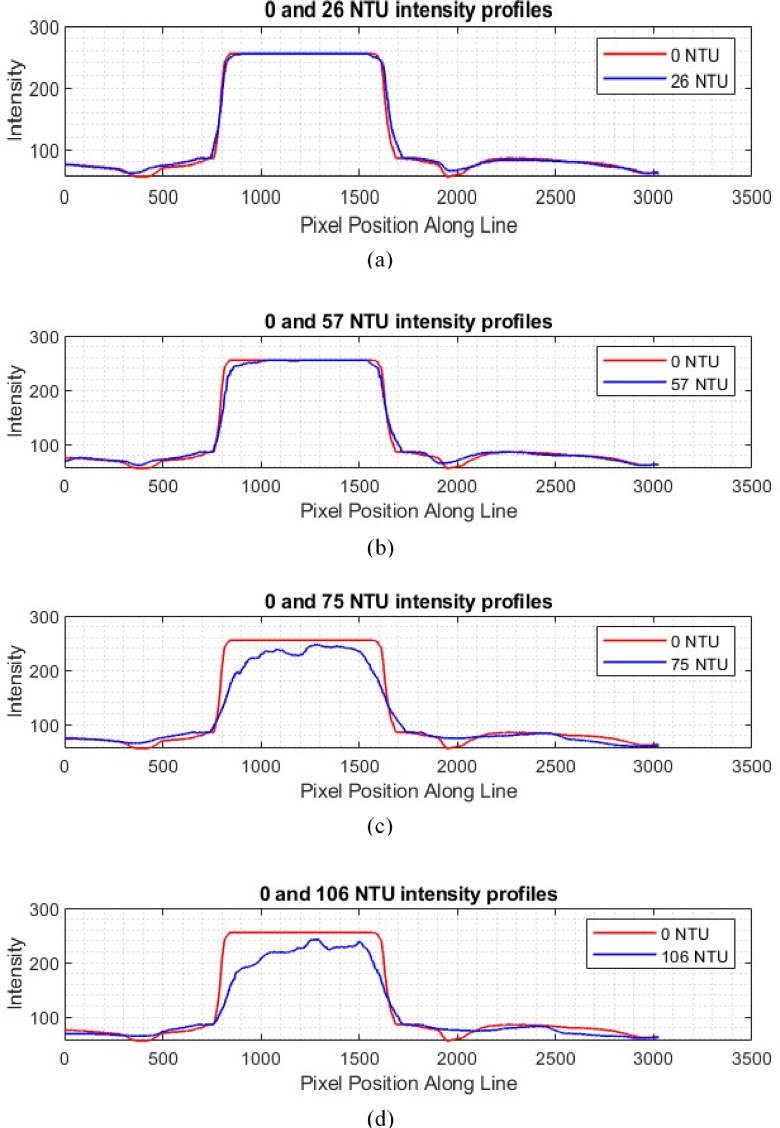
Intensity profile comparisons of 0 NTU with (**a**) 26 NTU, (**b**) 57 NTU, (**c**) 75 NTU, and (**d**) 106 NTU, along the refracted laser line.

Human urine can exhibit varying turbidity due to the presence of bacteria, pus, blood, crystals, protein, and other substances. This section investigates how different turbidity levels affect the results of the smartphone-based laser refractometer. Samples were prepared by mixing food-grade coffee powder with water to simulate turbidity. Table 3 shows that turbidity does affect the results of the proposed method, with the effect becoming significant at around 57 NTU. It is noteworthy that the average turbidity level of human urine is approximately 30 NTU, as stated by^37^. Turbidity levels in urine can be reduced using a centrifuge or by allowing the sample to rest for 30 min to 1 h, enabling sediments to settle at the bottom of the container. As observed in Fig. 4, increased turbidity affects the visibility of the refracted laser line by disrupting the measurement of its length (refer to Fig. 5), highlighting a limitation of the proposed device when used with samples exhibiting higher NTU levels. Figure 5 compares the intensity profiles of images obtained from samples with varying turbidity levels. It points out the differences in intensity profiles of samples with higher NTU levels compared to a reference sample with 0 NTU, indicating that image noise increases with turbidity.

The 12-pixel drop from 826.67 to 814.67 when moving from 0 NTU to 57 NTU, as noted in Table 3, reflects a reduction in signal intensity due to increased scattering and noise, as evident in the intensity profiles (e.g., Fig. 5b vs. 5a). It is noted that a 12-pixel drop corresponds to approximately 57.6 mg/dL, suggesting a significant underestimation of glucose concentration at 57 NTU. Two hypothetical cases can be considered here to clarify this. In the first case, comparing 100 mg/dL and 150 mg/dL at 30 NTU, the 50 mg/dL difference at constant turbidity would likely result in a small difference in pixel intensity, once adjusted for turbidity effects, enabling accurate differentiation with proper calibration. In the second case, comparing 100 mg/dL at 57 NTU with 150 mg/dL at 30 NTU, the 57 NTU sample would be underestimated by approximately 57.6 mg/dL due to turbidity, while the 30 NTU sample at 150 mg/dL would more closely reflect the true value. These estimates depend on the system’s calibration and the nonlinear impact of turbidity. To mitigate interference, one may consider a correction factor which can be derived by normalizing the turbid profiles (e.g., 57 NTU) to the clear profile (0 NTU).

**Table 3:** Results of nephelometer (NTU) and smartphone-based laser refractometer (pixels).

Sample	Coffee powder (g)	Nephelometric turbidity unit (NTU)	Smartphone-based laser refractometer (pixels)			
			Image 1	Image 2	Image 3	Average
1	0	0	825	828	827	826.67
2	0.25	26	826	827	826	826.33
3	0.50	57	816	814	814	814.67
4	0.75	75	-	-	-	-
5	1.00	106	-	-	-	-
6	1.25	131	-	-	-	-
7	1.50	148	-	-	-	-
8	1.75	179	-	-	-	-
9	2.00	190	-	-	-	-
10	2.25	228	-	-	-	-

### Assessing the effects of urine shelf-life

To ensure that the shelf-life of the samples does not affect the results, this test examined the outcomes of analyzing samples at different time intervals. Samples were measured using the proposed device on the day of acquisition, after 1 day, and after 2 days. For each measurement, three images were taken to calculate the mean reading. Results shown in Table 4 indicate that the standard deviation of results over the three days ranged from 0.19 to 6.30. The samples were refrigerated and stored in airtight screw-cap containers to prevent oxidation. Oxidation can alter the chemical, biological, physiological, and physical characteristics of urine through urea decomposition, creatinine alteration, protein breakdown, or colorimetric changes^38^. For example, creatinine alteration may change the urine concentration and thus affect the refractive index^38^. Because the samples from PBP were tested using a conventional glucometer, specific parameters such as proteins, ketones and bilirubin were not obtained. If present or elevated, these parameters could affect the refractive index of the urine as discussed further in Section “Comparison with fasting glucose levels”, which may explain the higher standard deviation observed in sample c16 (refer to Table 4).

**Table 4:** Results after 5 h, 1 day and 2 days of sample acquired on 2nd and 23rd August 2024.

Sample No.	mg/dL	Smartphone-based laser refractometer (pixels)				
		After 5 h	After 1 day	After 2 days	Mean	Std dev
c17	81	867.00	867.33	867.00	867.11	0.19
c3	84.6	868.33	866.00	868.33	867.56	1.35
c9	84.6	863.33	865.33	865.33	864.67	1.15
c1	86.4	862.67	863.33	865.00	863.67	1.20
c4	86.4	862.67	863.33	862.67	862.89	0.38
b11	100.8	867.00	866.33	867.67	867.00	0.67
b5	113.4	865.00	866.67	865.67	865.78	0.84
c10	117	860.67	860.67	861.00	860.78	0.19
c12	118.8	860.00	860.33	860.33	860.22	0.19
b1	120.6	845.00	842.67	847.67	845.11	2.50
b9	122.4	851.33	850.67	851.67	851.22	0.51
b4	126	848.33	849.33	848.00	848.56	0.69
b2	133.2	848.33	849.33	848.00	848.56	0.69
c18	135	850.33	850.33	845.00	848.56	3.08
c5	142.2	843.33	842.00	847.67	844.33	2.96
c21	142.2	842.00	841.67	843.00	842.22	0.69
c7	156.6	842.33	839.67	843.67	841.89	2.04
b7	176.4	846.67	847.67	845.67	846.67	1.00
c16	180	850.67	838.67	841.33	843.56	6.30
c19	180	837.33	836.00	839.33	837.56	1.68
b10	205.2	838.67	839.67	840.00	839.44	0.69
b16	234	833.33	832.67	835.00	833.67	1.20
b14	279	833.00	833.67	830.67	832.44	1.58
c14	286.2	831.67	831.67	832.67	832.00	0.58
c8	349.2	824.67	825.67	827.00	825.78	1.17
b8	487.8	818.33	819.00	818.67	818.67	0.33

### Comparison with fasting glucose levels

**Fig. 6:**
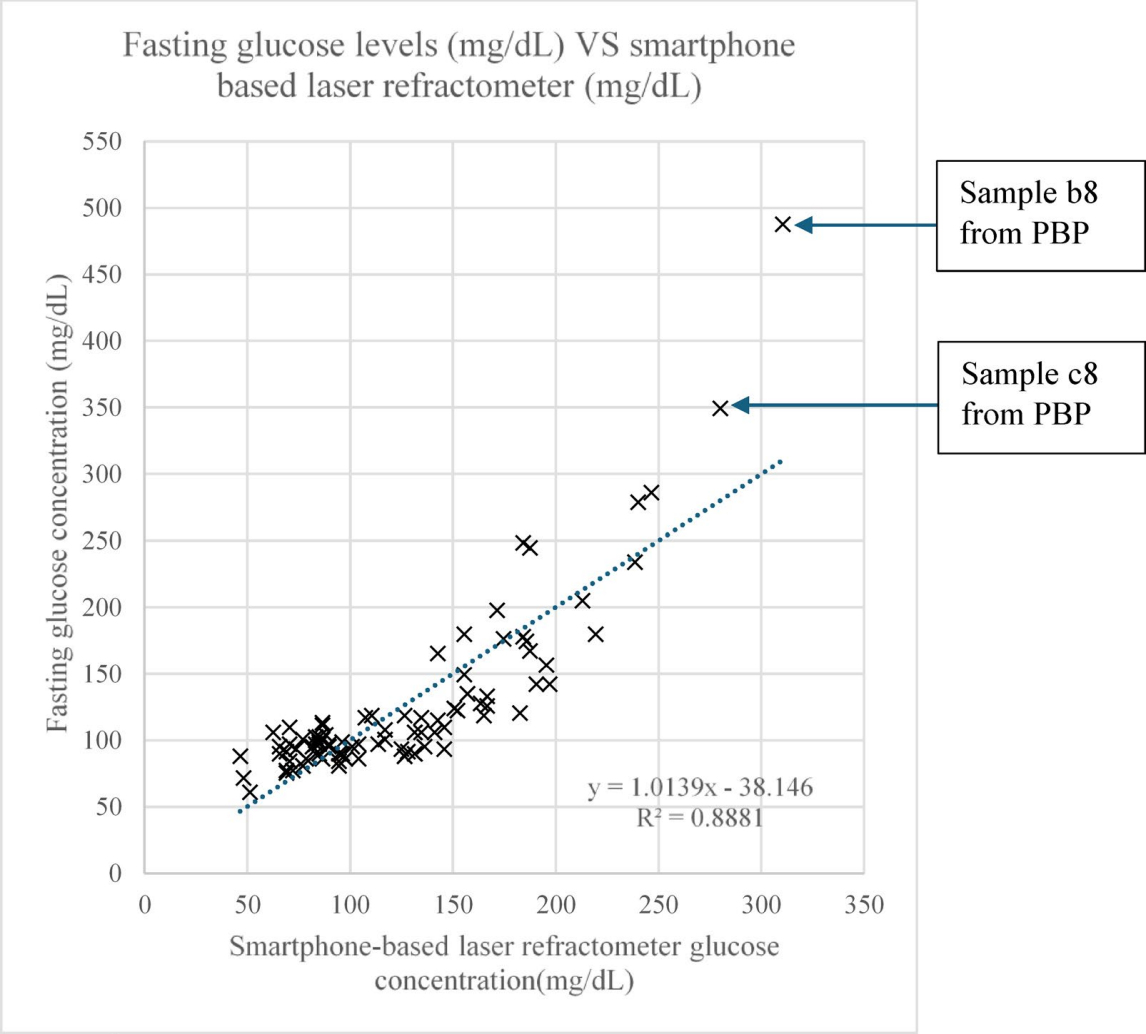
Result of fasting glucose concentrations (mg/dL) VS smartphone-based laser refractometer (mg/dL).

The first comparison involved 93 fasting glucose level samples from Advanced Pathology (M) Sdn. Bhd. (APSB) and Puncak Borneo Prison (PBP). The raw data showed promising results for the proposed method, with the highest standard deviation recorded at 5.86 (Refer to Appendix B for detailed mean and standard deviation for each measurement). As shown in Fig. 6, the comparison yielded a correlation coefficient of R^2^ = 0.8881 and a sensitivity of 4.8 mg/dL. It is important to note that bilirubin (a byproduct of hemoglobin), ketones (a byproduct of fat metabolism), and proteinuria (presence of protein in urine) are in liquid form^39–41^ and can affect the refractive index of urine samples^42–44^. In some cases, bilirubin may crystallize, thereby increasing turbidity. In this study, 11 samples reported proteinuria, 3 reported ketones, and none reported the presence of bilirubin. Of the 11 proteinuria samples, only 2 were visually cloudy (Sample No. 4558 and 4671). Of the 3 ketone-positive samples, 2 were also cloudy (Sample No. 4628 and 4840f). Due to their high turbidity, these four samples were deemed unusable and were excluded from analysis. Additionally, one sample (Sample No. 4844) was also omitted due to a high concentration of both protein and ketones.

Of the 160 total samples collected, 67 were found to be slightly cloudy or cloudy, with NTU levels exceeding the detectability threshold of the proposed device. This reflects the realistic conditions and limitations encountered during data collection, thereby enhancing the study’s integrity by demonstrating the real-world applicability and performance of the proposed device. Cloudy urine can result from the presence of pus, bacteria, blood, crystals, protein, and other substances. However, 18 slightly cloudy samples (with turbidity levels below 57 NTU) remained viable for use, as shown in the turbidity assessment section. These findings highlight a limitation of the proposed method, as optimal performance requires clear or slightly cloudy samples that are free from bilirubin, protein, and/or ketones. This issue may be addressed using advanced digital image processing algorithms, which will be explored in future studies.

As shown in Fig. 6, some outliers are present in the data. It is plausible that these deviations originated from the PBP dataset, which uses a conventional glucometer that does not provide information on ketone, protein, or bilirubin levels that may influence results as previously discussed.

## Conclusion

In this study, a non-contact smartphone-based laser refractometer for glucose monitoring was developed. The refractive indices of urine, determined by the refracted length of the laser line, were correlated with fasting blood glucose concentrations. To validate the concept, preliminary tests using food-grade glucose samples were conducted, showing a high correlation coefficient of 0.93. The effects of varying sample volumes and urine shelf-life on the measurements were found to be insignificant. Without the use of advanced image processing, the system’s turbidity detection threshold reached up to 57 NTU. When tested with 93 fasting glucose samples, the device demonstrated a relatively high correlation coefficient of 0.89 and a sensitivity of 4.8 mg/dL. Compared to colorimetric and biosensor methods reported in the literature, the proposed refractometry system offers an inexpensive and repeatable approach to glucose monitoring, making it suitable for telemedicine applications in rural areas.

## Methods

### Theory

**Fig. 7:**
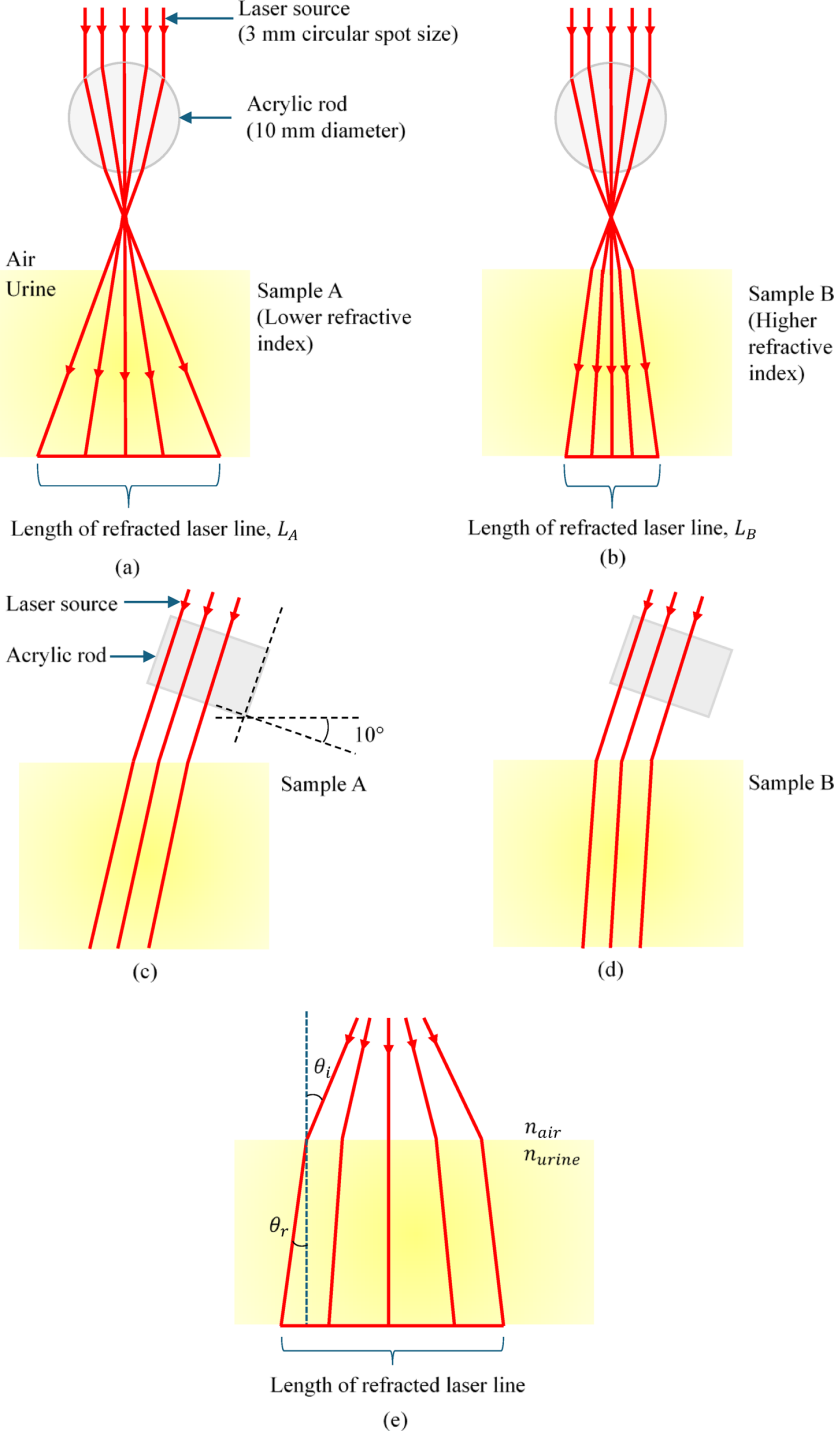
Front view of (**a**) Sample A with lower glucose concentration, (**b**) Sample B with higher glucose concentration. Side view of (**c**) Sample A with lower glucose concentration, (**d**) Sample B with higher glucose concentration. (**e**) Detailed front view of Sample B with higher glucose concentration. Images drawn not to scale.

In this study, the glucose concentration in urine is approximated based on the refractive index of the urine samples^45^, as described by Eq. (2). As illustrated in Figure 7, a circular laser beam passing diametrically through an acrylic rod undergoes total internal reflection, causing the beam to an elliptical shape. The size of the elliptical beam may with the diameter of the rod; however, the rod’s diameter remains constant throughout the experiment. At the air-urine interface, the size of the elliptical beam (defined by its minor and major axes) varies depending on the refractive index of the urine sample.

2 $$\:\frac{{n}_{air}}{{n}_{urine}}=\frac{\text{sin}{\theta\:}_{r}}{\text{sin}{\theta\:}_{i}}$$

where $\:{n}_{air}$ is refractive index of air, $\:{n}_{urine}$ is refractive index of urine, $\:{\theta\:}_{i}$ is angle of incident and $\:{\theta\:}_{r}$ is angle of refraction. Assuming that $\:{n}_{air}$ and $\:{\theta\:}_{i}$ are constants, $\:{\theta\:}_{r}$ is inversely proportional to $\:{n}_{urine}$. Hence, the length of refracted laser line is also inversely proportional to $\:{n}_{urine}$.

Assuming Sample B (Fig. 7b) has a higher refractive index than Sample A (Fig. 7a), the length of the refracted elliptical beam’s major axis in Sample B would be shorter than that in Sample A. Conversely, minor axis is comparatively larger in Sample A. Based on this observation, the glucose concentration in urine will be correlated with the major axis of the refracted elliptical beam, as changes in the major axis are more pronounced with varying refractive indices than those in the minor axis.

### Design and fabrication

**Fig. 8:**
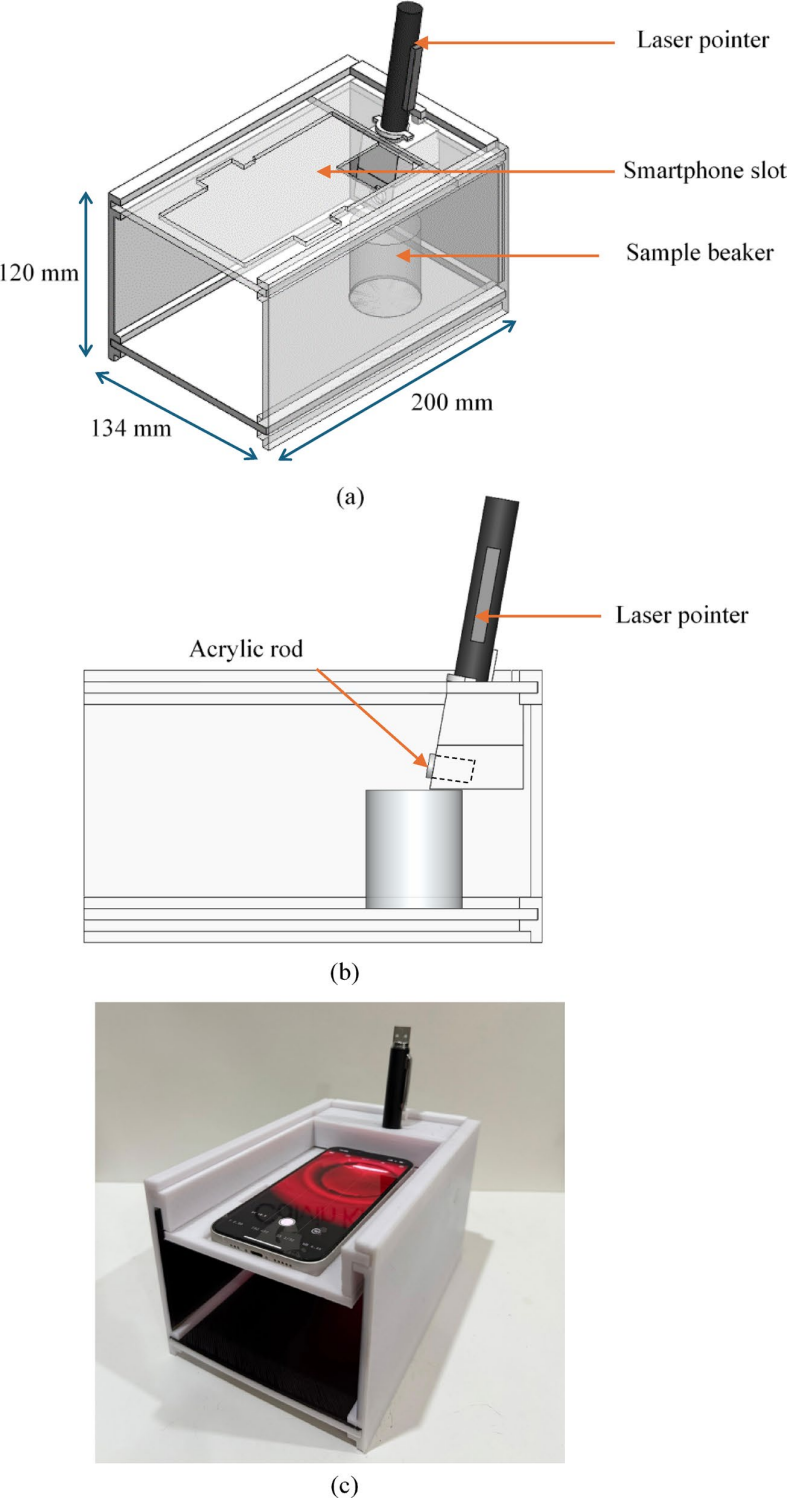
Smartphone-based laser refractometer design (**a**) in isometric and, (**b**) side view in AutoCAD. (**c**) 3D printed smartphone-based laser refractometer.

Figure 8a and b shows the proposed design of the smartphone-based laser refractometer. The laser beam is tilted at 10° to allow sufficient field-of-view the camera. The components are 3D-printed using PLA material with 20% infill. Figure 8c shows the actual system that consists of a rechargeable 5 mW laser pointer with a beam spot size of 3 mm at a standoff distance of 70 mm. To ensure consistent brightness, the laser must be fully charged before each measurement. For image capture, the ‘mcamera’ app was installed on an Apple iPhone 13 from the Apple Appstore to maintain consistent settings throughout all measurements (ISO 7.6 K and shutter speed 1/16). Autofocus was also disabled to eliminate focusing effects.

### Materials

In the preliminary experiment, a series of glucose solutions were prepared using water and glucose powder (Glucolin™), ranging from 70 to 300 mg/dL (3.9–16.7 mmol/L), with 10 mg/dL increments. Glucolin™ is a commercial energy drink brand made from dextrose monohydrate, a medicinal glucose used as an energy source for the body. Glucolin™ was used instead of pure laboratory-grade glucose due to its accessibility and ease of use. It contains readily soluble glucose with minimal additives, making it a suitable alternative for preliminary testing without significantly affecting the measurable properties of glucose. A future study will involve respondents consuming Glucolin™ to better understand its physiological effects and potential pathological implications, since it is safe for consumption, unlike laboratory-grade glucose. The solutions were prepared by mixing 100 mL of water with 0.07 g of glucose powder (measured using a digital weighing scale with ± 0.01 g sensitivity) to obtain 70 mg/dL glucose concentration. This process was repeated to produce concentrations up to 300 mg/dL in 10 mg/dL increments. Multiple images of each sample were captured to calculate the average length of the laser line. This procedure was repeated for all calibration and measurement stages.

A turbidity study was also conducted to determine the laser detection limit of the smartphone camera. Samples with varying turbidity levels (0–228 NTU) were prepared by mixing 200 mL of water with 0–2.25 g of food-grade coffee powder, in 0.25 g increments. While adding coffee powder to water does increase the refractive index, the addition of 0.25 g to 200 mL of water results in an extremely minor change (0.075%). Therefore, its impact on the refractive index was considered negligible and not a cause for concern. Since human urine turbidity can vary due to the presence of bacteria, pus, blood, crystals, protein, and more, this section evaluates the influence of turbidity on the accuracy of the proposed setup.

To examine the effect of sample shelf-life, measurements were taken at different time intervals: on the day of acquisition, after 1 day, and after 2 days^29^. Oxidation of urine samples was minimized by storing them at approximately 4 °C, using airtight screw-cap containers, and adding chemical preservatives. Biological and chemical activities are significantly reduced at this temperature. Samples were refrigerated (between 1.7 °C and 3.3 °C), as freezing is not recommended due to potential alterations in the chemical composition. Oxidation must be avoided, as it can affect the chemical, biological, and physical (including refractive index) properties of urine^38^. To prevent oxidation, fresh samples were obtained weekly from the pathology lab and stored under the conditions mentioned. Measurements were taken 4 h, 1 day, and 2 days after acquisition to assess stability over time.

The sample size for quantitative studies can be calculated using the following equation^46^;

4 $$\:sample\:size,\:N=\:\frac{{\left(Z\right)}^{2}{\left(Std\:Dev\right)}^{2}}{{\left(q\right)}^{2}}$$

where *Z* is the critical value and a standard corresponding value of confidence, and *q* is the margin of error. For a 95% confidence level, which is the common used confidence level, the critical value would be 1.96^46^. As for the standard deviation of this study it is 5.86 and the targeted margin of error is 1 mmol/L (18 mg/dL). Hence, the number of samples for this study can be calculated as follows;

$$\:sample\:size,\:N=\frac{{\left(1.96\right)}^{2}{\left(5.86\right)}^{2}}{{\left(1\right)}^{2}}=132$$

Hence, the minimum number of samples required for this study is 132 samples.

**Fig. 9:**
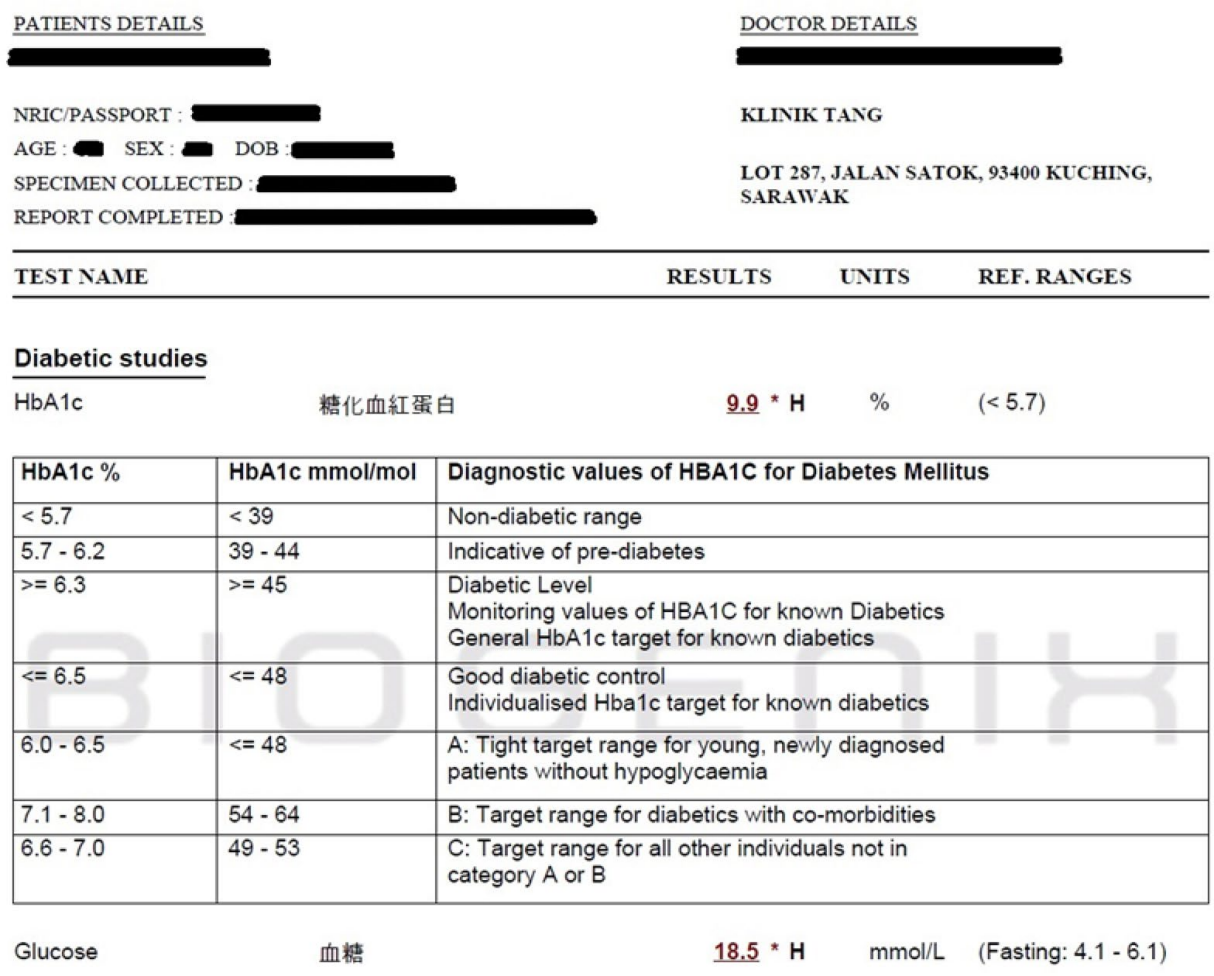
Blood report of patient.

A total of 123 and 37 actual urine samples were provided by (APSB) and (PBP), respectively. From APSB, urine samples were accompanied by urinalysis and blood reports using blood obtained by APSB as well. Urinalysis at APSB was conducted using the H-500 Urine Analyzer by Dirui Industrial Co., which provides fasting glucose level results. Test strips with multiple reagent pads were used to react with various substances in the urine. Optical sensors then detected color changes in the reagent pads, which correspond to glucose, protein, pH value, and specific gravity. For blood reports, blood samples were obtained by APSB where two glucose readings were recorded for each sample: fasting glucose levels and HbA1c. Blood samples were analyzed using the Blood Biochemistry Analyzer by Roche. These samples were mixed with reactive reagents for photometric, turbidimetric, and electrochemical detection, yielding results for glucose levels, HbA1c, bilirubin, creatinine, cholesterol, and protein. Figure 9 shows an actual blood report provided by APSB.

From PBP, urine samples and corresponding glucometer readings were obtained. Samples from 37 patients were collected in the morning on the same day, and their fasting glucose levels were measured using a conventional glucometer (Contour Plus Elite by Contour Plus). Out of the 123 samples from APSB, 56 included fasting glucose data, 15 included HbA1c data, and 52 included both fasting and HbA1c values.

## Supplementary Information

Below is the link to the electronic supplementary material.

Supplementary Material 1

Supplementary Material 2

## Data Availability

All data supporting the findings of this study are available within the paper and its Supplementary Information.
